# Glomerular cell crosstalk

**DOI:** 10.1097/MNH.0000000000000221

**Published:** 2016-03-25

**Authors:** Rachel Lennon, Salman Hosawi

**Affiliations:** a Wellcome Trust Centre for Cell-Matrix Research, Faculty of Life Sciences; b Institute of Human Development, Faculty of Human Sciences, University of Manchester; c Department of Paediatric Nephrology, Central Manchester University Hospitals NHS Foundation Trust (CMFT), Manchester, UK; d Department of Biochemistry, Faculty of Science, King Abdulaziz University, Jeddah, Saudi Arabia

**Keywords:** cellular crosstalk, extracellular matrix, extracellular vesicles, growth factors, signalling peptides

## Abstract

**Purpose of review:**

Glomerular filtration occurs in specialized, microscopic organelles. Each glomerulus contains unique cells and these cooperate to maintain normal filtration. Phenomenal adaptation is required for the glomerulus to respond to variable mechanical loads and this adaptation requires efficient communication between the resident cells. This review will focus on the latest discoveries related to signalling events that mediate the crosstalk between glomerular cells, and detail how disease processes can influence normal regulation.

**Recent findings:**

New data indicate that the crosstalk between glomerular cells involves an increasing number of secreted signalling ligands that act in an autocrine or paracrine fashion. Furthermore, extended roles for some of the classical signalling molecules have been described and there is emerging evidence of therapeutic strategies to manipulate cellular crosstalk. The glomerular extracellular matrix harbours many of these signalling ligands, acting as a reservoir and presenting ligands to cell surface receptors. Signals can also be transferred between cells by extracellular vesicles and this is an emerging concept in cellular crosstalk.

**Summary:**

Recent discoveries are building our understanding about glomerular cell crosstalk, and this review focuses on growth factors and signalling peptides, methods of delivery to target cells, and the potential for developing new therapies for glomerular disease.

## INTRODUCTION

**FIGURE 1:**
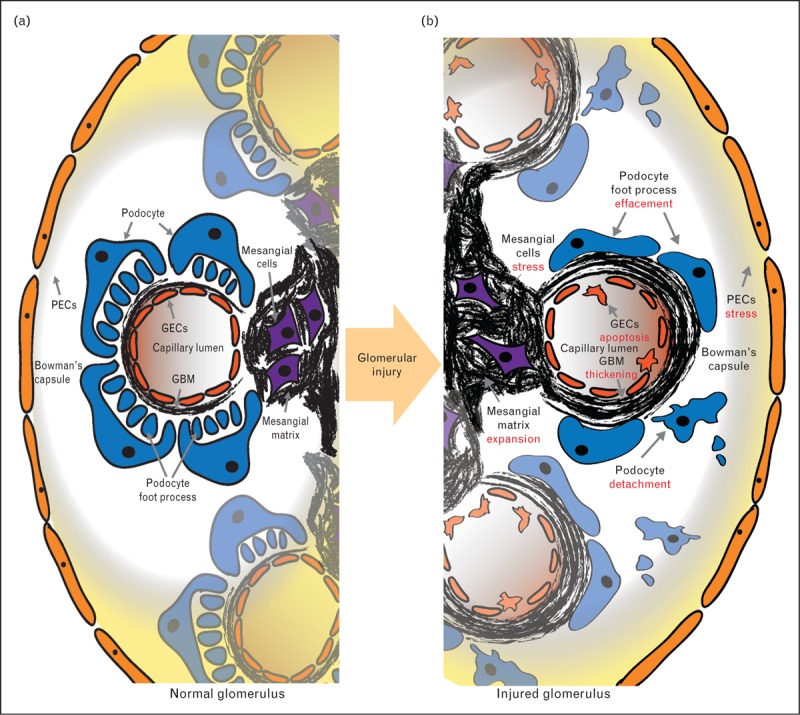
Glomerulus in health and disease. Four predominant cell types and their associated matrices form the unique glomerular structure. PECs line Bowman's capsule, podocytes, GECs form the capillary wall, and mesangial cells are centrally located within the glomerular tuft. The two main extracellular matrix compartments are the GBM and the mesangial matrix (a). In glomerular injury morphological change is observed across the spectrum of glomerular disease and includes: mesangial cell proliferation, mesangial matrix expansion, GBM thickening, podocyte foot process effacement, and podocyte detachment (b). GBM, glomerular basement membrane, GECs, glomerular endothelial cells; PECs, parietal epithelial cells.

The specialized function of glomerular filtration requires collaborative input from the four predominant cell types in the glomerulus. Each cell type plays a unique role and this is dictated by their molecular profile, morphology, and cellular location (Fig. 1). 

Mesangial cells occupy a central position in the glomerular capillary tuft where they secrete a distinct extracellular matrix (ECM) [1]. These cells play a key role in establishing and preserving glomerular capillary structure [2] and contributing to the regulation of glomerular filtration [3]. Mesangial cell expansion and increased ECM deposition is a signature of glomerular injury and this occurs across a wide spectrum of glomerular disease, from inflammatory insults [4] to systemic disease such as diabetes mellitus [5].

The glomerular capillary wall acts as a filtration barrier and exhibits selective permeability. The barrier comprises an inner layer of glomerular endothelial cells (GECs) facing the capillary lumen; visceral epithelial cells or podocytes are on the outer surface facing the urinary space, and a specialized ECM is flanked and produced by the two cell types. GECs are notably flat cells with abundant fenestrations that facilitate the function of filtration [6]. Podocytes are terminally differentiated cells, distinguished by their large cell bodies, long major processes, and smaller foot processes that interdigitate with other podocytes to cover the capillaries. Foot processes effacement, podocyte detachment, and apoptosis are hallmarks of glomerular injury [7].

Between the GECs and podocytes layers, there is a condensed network of ECM; the glomerular basement membrane (GBM). Typical of most basement membranes, the GBM comprises collagen IV, laminins, nidogens, and heparan sulphate proteoglycans [1]. However, specific isoforms of these proteins exist within the GBM and these are required for long-term maintenance of the glomerular filtration barrier. Genetic mutations in these key ECM isoforms result in human disease characterized by persistent proteinuria and progression to kidney failure [8,9].

Bowman's capsule surrounds the tuft of glomerular capillaries, and its innermost layer is formed by parietal epithelial cells (PECs) [10]. From a developmental perspective, both PECs and podocytes are derived from mesenchymal cells; however, the cell types diverge during the later stages of glomerular development. The PEC layer is adjacent to the epithelial cells of the proximal tubules and although the exact function of PECs is unclear, their potential involvement in replenishing the podocyte population has been proposed [10].

**FIGURE 2:**
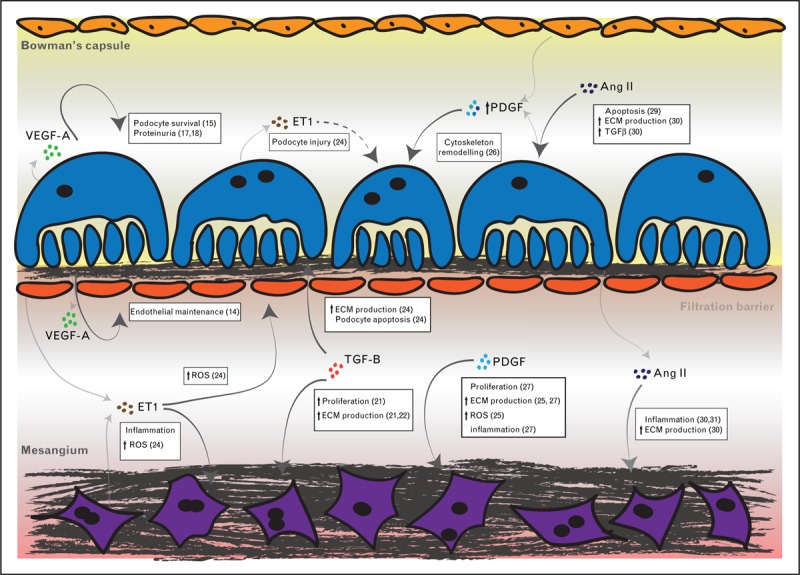
Action of secreted signalling ligands in the glomerulus. The effects of cellular crosstalk between parietal epithelial cells in Bowman's capsule, podocytes, and glomerular endothelial cells in the filtration barrier, and mesangial cells are illustrated. Relevant references are indicated in parentheses. Ang II, angiotensin II; ET-1, endothelin-1; ECM, extracellular matrix; PDGF, platelet-derived growth factor; ROS, reactive oxygen species; TGF-β, transforming growth factor β; VEGF-A, vascular endothelial growth factor-A.

For glomerular cells to function as an integrated filtration unit, cell–cell communication or crosstalk is required. Secreted growth factors and signalling peptides act as crosstalk effector molecules by engaging their target receptors and inducing signal transduction (Fig. 2). Secreted signalling ligands can have an autocrine effect on the same cell type or paracrine effect on nearby cells [11]. ECM plays an important role in storing secreted ligands, creating concentration gradients, and presenting ligands to cell surface receptors [12]. In addition to growth factors and signalling peptides, there is growing awareness of the role of extracellular vesicles that also facilitate cell crosstalk [13]. This review will focus on recent developments in our understanding of crosstalk in the context of glomerular health and disease.

**Box 1:**
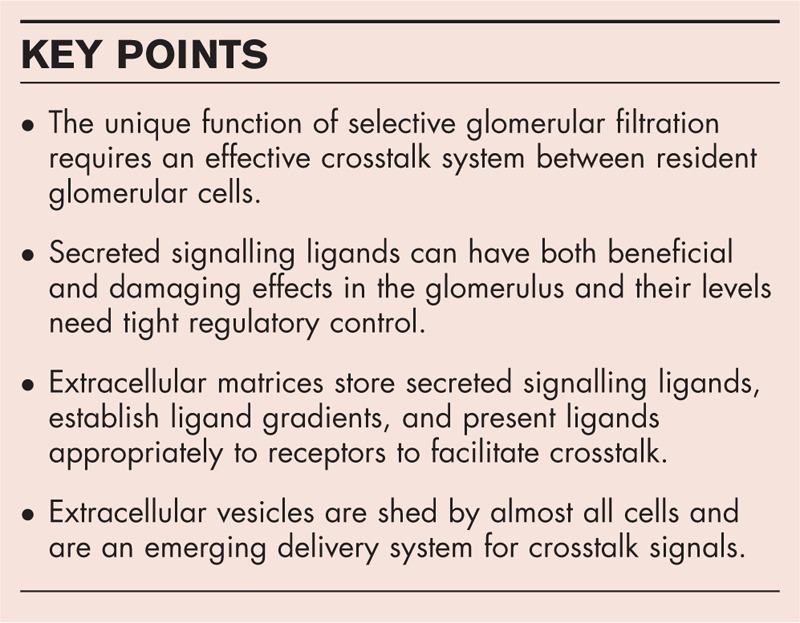
no caption available

## SECRETED GROWTH FACTORS AND SIGNALLING PEPTIDES

Vascular endothelial growth factor A (VEGF-A) is highly expressed in podocytes and its paracrine action on the adjacent endothelium is a fundamental requirement for intact glomerular function [14]. Increased VEGF-A levels are associated with a range of glomerular diseases, including diabetic nephropathy; however, whether this increase is a protective response against glomerular injury or the main driver of injury remains unclear. This concept was further investigated in a puromycin aminonucleoside nephrosis rat model of reversible glomerular injury [15^▪^]. The authors demonstrated a link between VEGF-A and Akt survival signalling via Gα-interacting, vesicle-associated protein (GIV). Puromycin aminonucleoside nephrosis increased GIV expression and following phosphorylation of GIV by vascular endothelial growth factor receptor 2 (VEGFR2), there was activation of Gαi3 and increased Akt2 and mammalian target of rapamycin complex 1(mTORC1) signalling. The authors proposed that this signalling complex could be targeted therapeutically to enhance podocyte survival.

VEGF-A isoforms were also further investigated in the context of diabetic nephropathy [16^▪▪^]. Increased expression of the VEGF-A_165_b isoform was found in patients with diabetic nephropathy and well preserved kidney function, suggesting a protective role for this isoform. This observation was recapitulated in a mouse model of VEGF-A_165_b overexpression. Furthermore, the systemic delivery of recombinant VEGF-A_165_b protected podocytes and GECs from apoptosis, and restored the endothelial glycocalyx in diabetic nephropathy mouse models. These studies were extended to include isolated glomeruli, and incubation with recombinant VEGF-A_165_b was associated with reduced permeability in human glomeruli from patients with diabetic nephropathy, raising the prospects of a therapeutic application for this protective VEGF-A isoform.

Following secretion by podocytes, VEGF-A can accumulate in the GBM and this feature was investigated in the context of Alport syndrome [17]. Using renal biopsy samples from Alport syndrome patients (*n* = 25) and controls (*n* = 11), the expression patterns of VEGF-A, VEGFR2 and the phosphorylation of VEGFR2 were studied using immunohistochemistry and immune electron microscopy. The authors found an increase in VEGF-A in the GBM of patients with Alport syndrome and this finding, together with the glomerular expression of phosphorylation of VEGFR2, correlated with the degree of proteinuria. As such, this study reports an association between high VEGF-A levels and glomerular injury; however, it is not clear whether the increased growth factor levels were also associated with increased podocyte survival signalling.

The promoter region of VEGF-A contains binding sites for numerous transcription factors and these include activating protein 1, which was investigated in the context of podocyte apoptosis in diabetic nephropathy [18]. In this study, silencing of VEGF-A or activating protein 1 reduced apoptosis and increased expression of the antiapoptotic protein Bcl-2. The same effect was seen with the use of VEGF-A inhibition. The authors proposed a protective effect of VEGF-A inhibition but acknowledged that this effect is likely to depend on the stage of glomerular injury; in early injury, VEGF-A may have a pathological role and this could explain the beneficial effect of early VEGF-A inhibition. With the progression of glomerular disease, VEGF-A levels are reduced and inhibition at the later stages may result in loss of the basal levels required to maintain the endothelium. Overall, these studies confirm the vital role of VEGF-A in maintaining glomerular function, and further support the notion that tight regulation of VEGF-A levels is required.

Transforming growth factor β (TGF-β) signalling is a central player in fibrosis, inflammation, cellular proliferation, and apoptosis [19]. These functions are mediated through distinct members of the TGF-β superfamily, including bone morphogenic proteins, growth differentiation factors, activins, and inhibins. In the context of renal fibrosis, the TGF-β/Smad signalling axis is a major driver of tubulointerstitial fibrosis and glomerulosclerosis. However, *Tgfb1*-null mice die from multiorgan inflammation within weeks [20], and therefore the effect of reducing this growth factor has not been fully evaluated. To determine the consequences of TGF-β dose manipulation, genetically titrated levels of TGF-β1 (10–300%) were generated in Akita diabetic mice [21^▪▪^]. The investigators found that lowering the expression levels of TGF-β1 in the glomerulus was associated with a reduction in features of diabetic nephropathy, including mesangial expansion, albuminuria, and reduced creatinine clearance. In contrast, an increase in TGF-β1 was associated with a more severe phenotype. These findings would support a therapeutic role for reducing glomerular or even podocyte-specific levels of TGF-β1.

TGF-β-activated kinase 1 (TAK1) is a known downstream effector of cytokine signalling involving TGF-β in addition to TNF-α and IL-1 and its activation leads to glomerular dysfunction. Podocyte-specific deletion of *Tak1* in the mouse led to a severe early phenotype with 50% of animals dying soon after birth. There was impaired formation of podocyte foot processes and prominent effacement. These findings were associated with proteinuria, increased VEGF-A production, and increased collagen deposition in the mesangial matrix [22], and therefore indicate that at least developmental inhibition of TAK1 is problematic.

The interplay between TGF-β and reactive oxidative species is being increasingly recognized as a perpetuating cycle in fibrosis [23]. In the context of glomerular injury, TGF-β1 was shown to upregulate mitochondrial NADPH oxidase 4, leading to podocyte apoptosis through the extracellular signal-regulated kinase 1/2 (ERK1/2) and mTORC1 signalling axis [23]. Subsequent inhibition of mTORC1 by the use of low-dose rapamycin protected podocytes, and there was an associated reduction in NADPH oxidase 4 expression, and oxidative stress induced apoptosis by TGF-β1. Focal segmental glomerulosclerosis is also associated with TGF-β activation in podocytes, and to gain mechanistic insight into this association, Daehn *et al.*[24^▪▪^] generated a podocyte-specific constitutively active TGF-β type I receptor mouse model to achieve activation of TFG-β signalling. The authors described increased endothelin-1 production by podocytes and also demonstrated mitochondrial oxidative stress in the adjacent GECs by a paracrine effect of endothelin-1. In turn, this led to podocyte apoptosis and this was rescued by the scavenging of reactive oxidative species. This study is a clear example of a perpetual cycle of injury involving crosstalk between GECs and podocytes.

In a similar manner to TGF-β, platelet-derived growth factor (PDGF) signalling is an important driver of glomerulosclerosis and renal fibrosis [25]. The PDGF family includes five members (PDGF-AA, AB, BB, CC, and DD) that differentially bind to their receptor PDGFR. The receptor itself is expressed as two isoforms (PDGFR-α and PDGFR-β). Within the glomerulus, mesangial cells express both isoforms and PDGFR-β is focally expressed by PECs. In a study of aged (27 month) mice, increased expression of PDGFR-β was observed in PECs and this was associated with an overall reduction in PECs density, increased sclerosis, and elevated levels of CD44, a marker of activated and profibrotic PECs [26]. PDGF is also known to induce the proliferation of mesangial cells, a feature of membranoproliferative glomerulonephritis and a mechanistic insight involving Erk5 and Akt activation was recently reported [27]. Rat mesangial cells were stimulated with PDGF leading to phosphorylation of the recently identified mitogen-activated protein kinase (MAPK), Erk5, and subsequent mesangial cell proliferation. A pharmacological inhibitor of Erk5 blocked this effect and the authors identified a positive feedback loop between Erk5, Akt, and PDGFR signalling. Erk5 may therefore represent a new target to block mesangial cell proliferation in membranoproliferative glomerulonephritis.

The renin–angiotensin system (RAS) plays an important role in the regulation of glomerular filtration. A cascade of vasoactive peptides acts systemically in addition to local RAS signalling between cells in the glomerulus [28]. Recent data suggest that renin and angiotensin I (Ang 1) have important roles in glomerular disease. Indeed, combined blockade of the prorenin receptor and angiotensin receptor 1 (ATR1) protected podocytes against apoptosis in a cell-based model of IgA nephropathy [29]. Furthermore, a novel link between angiotensin II (Ang II) and ECM accumulation in glomerulosclerosis was recently identified with the transcription factor sterol regulatory element-binding protein 1 (SREBP-1) [30^▪^]. In cultured mesangial cells treatment with Ang II led to increased SREBP-1 via AT1R. Furthermore, endoplasmic reticulum stress was a key element of SREBP-1 activation and inhibition of SREBP-1 prevented the upregulation of TGFβ both *in vitro* and *in vivo*, thus representing another potential candidate for targeted therapy. In another study, a common approach to targeting multiple RAS genes to provide greater therapeutic effect was investigated and this led to the unexpected connection between the Wnt/β-catenin signalling cascade and RAS signalling components [31^▪^]. The findings of this study suggest that the inhibition of the Wnt/β-catenin signalling can suppress multiple RAS genes and may therefore be a novel strategy to prevent the progression of glomerular disease.

## ROLE OF THE EXTRACELLULAR MATRIX

Throughout the body the ECM provides a structural scaffold to support cells. Condensed sheets of ECM form basement membranes and these underlie all epithelial and endothelial sheets. In addition to providing a structural scaffold, the ECM is rich in secreted growth factors and signalling peptides [32]. These ligands diffuse through the ECM and are presented to cell surface receptors to affect their autocrine or paracrine action. In our own proteomic investigations, we identified over 140 structural, regulatory, and secreted signalling proteins in the glomerular ECM [1]. To determine the cellular origin of ECM components, we studied cell-derived ECMs from podocytes and GECs. Here were found shared and distinct ECM components and by studying podocyte and GEC cocultures, we further revealed evidence of glomerular cell crosstalk in the assembly of the ECM [33]. Therefore, glomerular cells cooperate to assemble the ECM and the ECM itself serves as reservoir for signalling molecules to maintain glomerular function. This homeostasis is disrupted in glomerular injury and at a very early stage of this process we identified altered global ECM composition, with changes in netrin 4, fibroblast growth factor 2, tenascin C, collagen 1, meprin 1-α, and meprin 1-β [34]. The consequences of these changes in ECM composition on cell-matrix and cell-cell communication require further investigation.

## CROSSTALK BY EXTRACELLULAR VESICLES

Cell–cell communication mediated by extracellular vesicles is an emerging biological concept [35]. Extracellular vesicles are shed by almost all cells and are subclassified as exosomes and microparticles, according to their size. The potential roles for extracellular vesicles as disease biomarkers have been investigated in a number of glomerular diseases, including focal segmental glomerulosclerosis, diabetic nephropathy, and IgAN [35]. Extracellular vesicles may contain DNA, RNA, protein, and lipid components, and the role of these cargos in effecting downstream effects in the glomerulus is yet to be determined. However, there is more evidence of extracellular vesicles in the context of tumour metastasis. Tumour-derived exosomes have recently been shown to fuse with cells at the metastatic destination and this tissue preference was associated with the expression of integrin adhesion receptors in exosomes [36]. Subsequent targeting of the exosomal integrins led to a reduction in the uptake of exosomes and also a clinically relevant decrease in tumour metastasis. These data highlight the potential for exploiting extracellular vesicles not only as biomarkers but also as therapeutic targets to control pathogenic cellular crosstalk.

## CONCLUSION

There is now a wealth of data to support a role for cellular crosstalk between resident cells in the glomerulus. This review has focused on secreted signalling ligands and has shown extended roles for VEGF, TGF-β, PDGF, and RAS components. The role of the ECM in creating a reservoir of signalling ligands was introduced and a greater understanding of this complex extracellular niche will help to further define mechanisms of cell–cell communication. The emerging role of extracellular vesicles, capable of delivering specific signalling ligands and downstream effects, was also introduced, and this rapidly growing area of research may soon impact on our understanding of glomerular cell crosstalk. Although the main focus has been on disease, there is additional evidence to support a role for glomerular crosstalk in the development and in the normal regulation of glomerular function.

## Acknowledgements

None.

### Financial support and sponsorship

The work was supported by a Wellcome Trust Intermediate Fellowship award (090006) to R.L. and a PhD studentship award to S.H. from The Ministry of Higher Education, Kingdom of Saudi Arabia (K1199).

### Conflicts of interest

There are no conflicts of interest.

## REFERENCES AND RECOMMENDED READING

Papers of particular interest, published within the annual period of review, have been highlighted as:

▪ of special interest
▪▪ of outstanding interest
